# Air pollution exposure—the (in)visible risk factor for respiratory diseases

**DOI:** 10.1007/s11356-021-13208-x

**Published:** 2021-03-04

**Authors:** Gabriel-Petrică Bălă, Ruxandra-Mioara Râjnoveanu, Emanuela Tudorache, Radu Motișan, Cristian Oancea

**Affiliations:** 1 Department of Pulmonology, University of Medicine and Pharmacy “Victor Babeș”, P-ța Eftimie Murgu nr.2, Timișoara, 300041 Timiș Romania; 2 Department of Pulmonology, University of Medicine and Pharmacy “Iuliu Hațieganu”, Cluj-Napoca, Romania; 3 Magnasci SRL, Timișoara, Romania

**Keywords:** Air pollution, Particulate matter, Exposure, Respiratory diseases, Chronic obstructive pulmonary disease, Lung cancer, Asthma

## Abstract

There is increasing interest in understanding the role of air pollution as one of the greatest threats to human health worldwide. Nine of 10 individuals breathe air with polluted compounds that have a great impact on lung tissue. The nature of the relationship is complex, and new or updated data are constantly being reported in the literature. The goal of our review was to summarize the most important air pollutants and their impact on the main respiratory diseases (chronic obstructive pulmonary disease, asthma, lung cancer, idiopathic pulmonary fibrosis, respiratory infections, bronchiectasis, tuberculosis) to reduce both short- and the long-term exposure consequences. We considered the most important air pollutants, including sulfur dioxide, nitrogen dioxide, carbon monoxide, volatile organic compounds, ozone, particulate matter and biomass smoke, and observed their impact on pulmonary pathologies. We focused on respiratory pathologies, because air pollution potentiates the increase in respiratory diseases, and the evidence that air pollutants have a detrimental effect is growing. It is imperative to constantly improve policy initiatives on air quality in both high- and low-income countries.

## Introduction

Air pollution represents one of the biggest risk factors for human health. It is an invisible killer that hides around us, influencing both young and old generations. According to the World Health Organization (WHO), each year, 7 million people die due to air pollution. The most affected pathologies are chronic obstructive pulmonary disease, lung cancer, and respiratory infections, including pneumonia, stroke, and heart disease. Nine out of 10 individuals breathe air with polluted compounds, which penetrate deep into the lung tissue, and furthermore in the cardiovascular system (Ghebreyesus 2018) (World Health Organization 2018) (Tiotiu et al. 2020).

The most exposed individuals are elderly persons, infants, pregnant women, and persons with comorbidities (Mannucci et al. 2015). An estimated 43% of lung diseases and 24% of strokes are attributed to air pollution.

We performed an electronic search on PubMed for literature published in the last 5 years, with the last search date on February 15, 2020. The following terms were used: “air pollution,” “particulate matter,” “biomass,” “smoke,” “sulfur dioxide,” “nitrogen dioxide,” “carbon monoxide,” “ozone,” “chronic obstructive pulmonary disease,” “asthma,” “lung cancer,” “idiopathic pulmonary fibrosis,” “respiratory infections,” “bronchiectasis,” and “tuberculosis.” The terms were also searched in combination, such as “particulate matter and lung cancer” and “air pollution and chronic obstructive pulmonary disease.” The results were restricted to full-text studies of humans and mechanisms via animal experiments. Systematic reviews, meta-analyses, reviews, and publications from the WHO were included in the search. We additionally included older references if they had an important impact on the subject area, according to our knowledge.

### General air pollution

Air pollutants are classified into two main categories: primary air pollutants (pollutants emitted directly into the atmosphere) and secondary air pollutants (pollutants that are formed within the atmosphere itself) (World Health Organization 2005) (Mannucci et al. 2015).

Primary air pollutants are those released from a direct source, such as exhaust pipes from a mobile vehicle, or from a stationary source, such as factory chimneys. At the same time, contaminated dust can also be distributed by the wind to uncontaminated areas. These pollutants can be calculated by measuring the amounts emitted by the source itself. Primary air pollutants are represented by oxides of nitrogen, carbon monoxide (CO), sulfur dioxide (SO_2_), volatile organic compounds (VOCs), and carbonaceous and non-carbonaceous primary particles. The International Agency for Research on Cancer (IARC) has classified emissions from burning coal in an indoor environment as potentially carcinogenic to humans. These were observed with sufficient evidence in both animals and humans (Barone-Adesi et al. 2012).

There are many sources of primary air pollutants, but the most significant are road traffic and power plants. Additionally, industrial and residential heating based on wood, coal. or oil contributes to increasing the degree of air pollution (World Health Organization 2005) (Guarnieri and Balmes 2014) (Kravchenko and Lyerly 2018) (Minichilli et al. 2019).

Secondary air pollutants are formed through chemical reactions in the atmosphere, with natural components such as water and oxygen. Secondary air pollutants include ozone (O_3_), oxides of nitrogen, and particulate matter (PM) (World Health Organization 2005) (Guarnieri and Balmes 2014) (Mannucci et al. 2015)

The chemical composition of air pollutants is diverse and depends on the source. Additionally, a seasonal pattern is observed, with higher average daily concentration levels of nitrogen dioxide (NO_2_), CO, PM10, and fine particulate matter (PM2.5) during the cold season, while O_3_ concentration levels tended to be higher during the warm season (Bernardini et al. 2019)

Air pollutants released by coal-fired power plants have raised concerns about their impact on public health. PM2.5 can have both short-term and long-term consequences on human health. In a study conducted by Cheng-Kuan Lin et al., a strong association was observed between the increase in coal capacity per person and an increase in the relative risk for lung cancer, both in men and women. These were observed by a factor of 85% among women and 59% among men. Based on these data, it is predicted that in 2025, a total of 1.37 million cases of lung cancer will be correlated with coal-fired power plants (Lin et al. 2019).

## Outdoor (ambient) air pollutants

### Sulfur dioxide

Sulfur dioxide (SO_2_) and PM come from the process of burning fossil fuels and represent the essential components of air pollution. Sulfurous and sulfuric acids are formed as a result of the oxidation process of SO_2_. Natural sources include volcanoes, but significant concerns have been encountered in large metropolitan regions where coal is being used for domestic heating or for poorly controlled combustion for industrial installations (World Health Organization 2005).

Exacerbation of respiratory symptoms has been shown to be related to exposure to SO_2_ emitted by coal-burning power plants, and lower concentrations were associated with respiratory deaths. The major anthropogenic sources of SO_2_ are found in developing countries and come from burning fossil fuels that contain sulfur. The reason for burning fossil fuels is due to heating homes, use in power plants, and powering vehicles (Kravchenko and Lyerly 2018). Sulfur dioxide concentrations are lower since indoor concentrations are absorbed by walls, furniture, and inhalation systems (World Health Organization 2005).

### Nitrogen dioxide

There are many species of nitrogen oxides, but the one with the most important effect on human health is NO_2_. NO_2_ is a gas with a brown color, having a distinctive powerful scent. Nitric oxide spontaneously produces dioxide when it is exposed to air. It is a powerful oxidant that produces nitric acid and nitric oxide by reacting with water, and it is an important trace gas affecting human health. It absorbs solar radiation, contributing to low visibility in the atmosphere and plays a direct role in global climate change.

NO_2_ undergoes further transformations, and after the photochemical reaction sequence is initiated by its solar radiation-induced activation, newly generated pollutants are created, containing organic, nitrate, and sulfate particles, all measured at PM2.5 and PM10. Among natural sources, the represented sources include lightning, inclusion of stratospheric nitrogen oxides, and bacterial and volcanic actions. The major anthropogenic sources are mobile sources (combustion engines) and stationary combustion sources (power generation sources) (World Health Organization 2005) (Kravchenko and Lyerly 2018)

Patients with asthma and chronic obstructive pulmonary disease (COPD) have been associated with an increased risk of respiratory hospitalization after exposure to NO_2_ (Kravchenko and Lyerly 2018). In addition, exposure to air pollution due to traffic vehicles increases the risk of developing bronchiolitis obliterans post-lung transplant syndrome (Johannson et al. 2015).

In China, a systematic review and meta-analysis by Sun et al. identified a positive correlation between short-term ambient exposure to NO_2_ and pulmonary diseases. A 10-μg/m^3^ increase in NO_2_ concentration was associated with an increase of 1.4% in mortality due to respiratory disease and 1.0% in hospital admission. Elderly individuals had an even higher susceptibility (Sun et al. 2017).

### Carbon monoxide

The most important source of environmental CO is incomplete combustion of traffic-related fossil fuels, leading to >50% of emissions in urban areas, other sources (such as manufacturing and natural processes, etc.) being less prominent (World Health Organization 2005).

Carbon monoxide is considered to be a “silent killer” due to its toxicity arising from its ability to bind hemoglobin more strongly than oxygen, increasing the risk of asphyxia-related deaths at high levels of exposure or hypoxic tissue damage at low levels of exposure (World Health Organization 2005).

Asthma, bronchiectasis, and pneumonia have been associated with ambient short-term exposure to CO (Zhao et al. 2019). The study of Zhao et al. conducted over 4 and 1/2 years, with a daily mean ambient CO of 0.88 mg/m^3^, varying from 0.40 to 3.13 mg/m^3^, reported an increased risk for daily outpatient visits for respiratory disease, with a higher effect on women and elderly patients (Zhao et al. 2019).

In different studies, a positive association between daily exposure to PM2.5, SO_2_, and CO and an increased risk of mortality from respiratory diseases and lung cancer was reported. For a 1-mg/m^3^ increase in CO and a 10-μg/m^3^ increase in PM10, there has been a 1.9% and 4.8% increase in total deaths, respectively (Xue et al. 2018) (Table 1).

**Table 1:** Associations between air pollutants and respiratory diseases

Authors	Disease	Air pollutant association	Ethnicity/nationality
Liang et al. (2019)	COPD	PM2.5	China
Huang et al. (2019)	COPD	PM2.5	Taiwan
Havet et al. (2019)	Asthma	PM10, O_3_	France
Cadelis et al. (2014)	Asthma	PM10, PM2.5–10	Caribbean
Akpinar-Elci et al. (2015)	Asthma	PM10, PM2.5–10	Caribbean
Guarnieri and Balmes (2014)	Asthma	PM2.5, PM 10	Meta-analyses
Xing et al. (2019)	Lung cancer	PM2.5, PM10, O_3_	China
Hamra et al. (2014)	Lung cancer	PM2.5	Meta-analyses
Gharibvand et al. (2017)	Lung cancer	PM2.5	USA, Canada
Wang et al. (2019a)	Lung cancer	PM2.5	China
Winterbottom et al. (2018)	IPF	PM10	USA
Johannson et al. (2018)	IPF	NO_2_, PM2.5, PM10	USA
Johannson et al. (2014)	IPF	O_3_, NO_2_	South Korea
Nsoh et al. (2019)	Respiratory infections	PM2.5	Cameroon
Z. Zhang et al. (2019)	Respiratory infections	PM2.5, PM2.5–PM10	China
Zheng et al. (2017)	Respiratory infections	PM10, NO_2_, SO_2_	China
Goeminne et al. (2018)	Bronchiectasis	PM10, NO_2_	UK
Garcia-Olivé et al. (2018)	Bronchiectasis	SO_2_	Spain
Popovic et al. (2019)	Tuberculosis	PM2.5	Asia, Europe, North America
Zhu et al. (2018)	Tuberculosis	PM10, NO_2_, SO_2_	China
Lai et al. (2016)	Tuberculosis	PM2.5	Taiwan
Jassal et al. (2012)	Tuberculosis	PM2.5	USA
Li et al. (2019)	Tuberculosis	PM2.5	China
Yao et al. (2019)	Tuberculosis	PM2.5, PM10, O_3_, CO	China

### Volatile organic compounds

Volatile organic compounds (VOCs) are compounds with a high vapor pressure of one or more carbon atoms, which will lead to their release in the atmosphere (Ciganek and Neca 2008). Compounds from the atmosphere, in a state of a vapor phase, such as oxygenates, hydrocarbons, halogenates, and other carbon compounds, are the main components of VOCs (World Health Organization 2005).

There are different sources of VOCs. They can arise from natural causes, such as forest fires, vegetation, and animals, but also from artificial causes, such as vehicles. Natural sources of VOCs represent a higher percentage, but anthropogenic sources contribute significantly to reducing air quality.

The most important sources of VOCs are released by industrial and agricultural sources. At the same time, handling solvents or solvent-based products contributes significantly to VOC concentrations. Samples that were collected from road dust or soil were based on volatile organic compounds, such as benzene, toluene, styrene, ethylbenzene, and xylene, as well as aliphatic hydrocarbons (mostly *n*-alkanes), dichloromethane, and disulfide carbon (Ciganek and Neca 2008).

Oxidative stress and decreased lung function are related to exposure to low levels of VOCs. Additionally, airway inflammation could be related to exposure to increased levels of VOCs in everyday life (Kwon et al. 2018).

In a national cross-sectional representative survey that was conducted by the Indoor Air Quality Observatory, *N*-undecane and 1,2,4-trimethylbenzene were correlated with asthma in 8.6% of cases, while trichloroethylene, ethylbenzene, and m/p- and o-xylene were associated with rhinitis (Billionnet et al. 2011).

In a French cross-sectional study on farmers, indoor mean VOC concentrations were smaller in workplaces than in dwellings. Working in a rural environment involves a degree of exposure to various risk factors such as agricultural machinery and fires, exposure to capricious weather, agricultural land working, and exposure to various organic compounds. Following this study, individuals mentioned that respiratory symptoms, such as dyspnea, cough, sneezing, and wheezing, were the most common. They were present in 44% of people when manipulating plants that had been harvested. Asthma and early airway obstruction were linked with exposure to VOCs and PM and in farmers (Maesano et al. 2019).

### Ozone

Ozone (O_3_) is a chemical compound that is not directly emitted into the air but is formed through a series of complex reactions. Atomic oxygen and nitric oxide are formed after NO_2_ splits. Atomic oxygen later combines with oxygen-forming ozone. Ozone is disintegrated by reacting with nitric oxide, resulting in NO_2_ and oxygen. Ambient concentrations depend on several factors: the concentration of NO_2_ and VOCs, sunshine intensity, atmospheric convection, and the proportion of VOCs to nitrogen oxides (World Health Organization 2005) (Guarnieri and Balmes 2014).

Daily concentrations of air pollutants are higher in the cool seasons than in the warm seasons, except for O_3_, which is higher during warm seasons (Wang et al. 2019d). Ground-level ozone (O_3_) is considered one of the most dangerous air pollutants in the USA and the European Union, being a strong oxidizing compound. In recent years, O_3_ levels have remained high without showing any decline and will remain a constant public health problem, especially with the progression of global warming (Guarnieri and Balmes 2014) (Wang et al. 2019c).

Concentrations of O_3_ can increase during late spring and summer months due to photochemical reactions, along with its precursors, such as VOCs. High concentrations of O_3_ can be associated with various local and long-range transports of anthropogenic emissions. In winter, lower photochemical processes result in a smaller contribution of this factor to PM2.5 mass (18%) (Bari and Kindzierski 2017). Human exposure to ozone is correlated with a high risk of respiratory disorders, such as asthma exacerbation and lung inflammation, loss of lung function, and cystic fibrosis (Johannson et al. 2014). Additionally, it has been shown to interact with cerebral blood vessels by modulating the expression of genes involved in brain vasoreactivity, irritating mucous membranes, altering the levels of serotonin, and affecting the immune system (Bernardini et al. 2019).

### Particulate matter

According to the World Health Organization (WHO), the standard for daily PM2.5 concentration is 25 μg/m^3^, while the annual average is 10 μg/m^3^. Approximately 92% of the world’s population lives in locations where the mean PM2.5 mass concentration surpasses this amount. Approximately 3 million persons die from outdoor air pollution each year (Wang et al. 2019b).

Particulate matter (PM) is represented by a complex mixture containing components with diverse physical and chemical characteristics. The potential for these particles to cause injury varies due to their chemical composition and source. Additionally, their size and physical characteristics represent major concerns for public health (World Health Organization 2005).

Particles are classified in general by their aerodynamic diameter. PM can generally be classified into three major fractions: coarse particles, exceeding 2.5 μm in aerodynamic diameter; fine particles, which are smaller than 2.5 μm; and ultrafine particles, which are smaller than 0.1 μm (100 nm). PM10 contains PM2.5 and thoracic coarse mass (the distinction between PM10 and PM2.5 is generally presented as coarse mass). In general, PM10 mass contains 40–90% of the PM2.5, the rest being considered coarse PM (World Health Organization 2005) (Guarnieri and Balmes 2014) (Mannucci et al. 2015).

PM with a 2.5–10-μm aerodynamic diameter, also known as coarse PM, is stored particularly in the head and in the upper respiratory tract. PM2.5 is usually stored in the deep respiratory airways, primarily in the small airways and alveoli, while ultrafine PM (<0.1μm) is stored in the alveoli (World Health Organization 2005) (Guarnieri and Balmes 2014) (Liang et al. 2019).

PM2.5 are primarily formed from gases. These particles usually emerge as ultrafine particles created by the formation of very small particles (nuclei) by condensation-nucleation of low vapor pressure substances generated by chemical reaction into the atmosphere or by high-temperature vaporization (World Health Organization 2005) (Mannucci et al. 2015) (Kravchenko and Lyerly 2018). The principal precursor gases are represented by nitrogen oxides, ammonia, SO_2_, and VOCs. At the same time, fluctuations in the concentrations of these compounds may alter ambient PM concentrations. On the days when PM10 concentration exceeds 50 μg/m^3^ PM, nitrate becomes the main compound of PM10 and PM2.5 (World Health Organization 2005)(Kravchenko and Lyerly 2018).

There are numerous sources of both human activity and natural source-related particles. Specific sources impact different regions of the world, but more than two-thirds of PM 2.5 is due to industrialization in developed areas.

The origin of PM2.5 is variable. It can come from several sources, such as vehicle traffic, followed by dust generation, aerosols from regional transport, agricultural activities, and the burning of biomass for cooking or heating. It is difficult to appreciate the contribution of each source and to further recognize the PM formation mechanism (Zhang and Cao 2015).

Photochemical conversion of secondary pollutants (SO_4_^2−^, NO_3_^−^, and NH4+) represents 3.7% of PM2.5 and 2.4% of PM10 (Lee et al. 2018). Concentrations of NH_4_^+^, NO_3_^−^, and SO_4_^2−^ on days with higher pollution can be two to four times higher than on unpolluted days (*p* < 0.01), according to the study of Pan et al, where PM2.5 samples were gathered from two metropolitan areas (Beijing and Shanghai) on polluted and unpolluted days in the fall of 2017 (Pan et al. 2019). Additionally, seasonal variations in PM2.5 have been described in different regions of the world.

In a Canadian study conducted by Md. Aynul Bari from May 2009 to December 2015, the overall mean and median concentrations of PM2.5 were comparatively higher in winter than in summer (Bari and Kindzierski 2017). The same observation was reported in the Chinese study of Yan-Lin Zhang et al., with remarkable seasonal variability in PM2.5, which was highest during winter and lowest during summer. On the other hand, increased levels of PM2.5 are also found in the spring and autumn due to the contribution of dust particles and the start of burning biomass. In addition, the lowest and highest PM2.5 concentrations frequently occur in the afternoon and evening hours (Johannson et al. 2014).

An investigation of the origin of wintertime high PM2.5 pollution days revealed that in addition to traffic emissions, another significant source that helped increase PM2.5 in winter was a mixed factor represented by local industry and agriculture (deduced as gas emission sources and upstream oil) (Bari and Kindzierski 2017).

## Indoor air pollutants

### Biomass smoke

Biomass smoke is a major public health problem (Balcan et al. 2016). Coal and biomass fuels are used by almost 3 billion people worldwide. A large part of the world’s population still depends on solid fuel for cooking, firewood, and charcoal (Nsoh et al. 2019). Individuals generally use different types of fuels for heating and cooking, such as “smoky coal” (bituminous), “smokeless coal” (anthracite), and wood (Barone-Adesi et al. 2012).

Compounds emitted from smoky coal combustion are present in abundance, such as polycyclic aromatic hydrocarbons (PAHs), methylated PAHs, and heterocyclic aromatic compounds containing nitrogen. Following an incomplete combustion process, solid fuel releases a significant amount of toxic particles. These will then be inhaled and cause multiple respiratory symptoms. Symptoms may include upper respiratory tract conditions, such as cough, nasal obstruction, vocal dysfunction, rhinorrhea, laryngeal spasm, and lower respiratory tract symptoms, such as dyspnea, wheezing, and cough (Nsoh et al. 2019).

Approximately half of the world’s population cooks and heats using unprocessed biomass fuels and coal. Several diseases are associated with exposure to solid fuel smoke, including lung cancer, chronic obstructive pulmonary disease, and respiratory infections (Barone-Adesi et al. 2012). In the study by Barone-Ades et al., it was observed that mortality due to lung cancer was higher in people who used smoked charcoal than in those who used no-smoke charcoal throughout their lives. In the study, 9962 people used smokeless coal, and 27,310 used smoked coal. The absolute risk of death from lung cancer in individuals who used smoked charcoal was higher for women (20%) than men (18%). As a comparison, the percentage of people who used smokeless charcoal and developed lung cancer was only 0.5%. These values are similar to those observed in heavy smokers in Western countries, with a value between 20 and 26% (Barone-Adesi et al. 2012).

Lung function begins to deteriorate after exposure to smoke for more than 15 years. The chance of having modified pulmonary function increases as the duration of exposure increases. In rural areas, women are generally more exposed due to their conventional lifestyle. In a case-control study, from a total of 115 women exposed to biomass smoke, 23.8% had small airway disease, 19.1% had obstruction, and 17.3% had a restriction pattern on pulmonary function tests (Balcan et al. 2016).

## Public health focus on respiratory disease

### Chronic obstructive pulmonary disease

COPD is a multifactorial condition characterized by chronic airway obstruction that is incompletely reversible, progressive, and associated with an abnormal inflammatory response of the lung to harmful particles or gases (Singh et al. 2019).

Ambient air pollution is associated with COPD morbidity and mortality. From systemic analyses, it was observed that morbidity from COPD is correlated with a short-term increase in air pollution (Adar et al. 2014)(Zhang et al. 2016) (Tian et al. 2018).

The body’s response may differ from person to person. The ability of each person to react to air pollution may differ in the Chinese population compared with the North American or European population due to differences in air pollution concentration and the composition of the polluted air. At the same time, the pre-existing pathology of one population may be different from another. In a study conducted in Beijing, China, a reduction in the average concentration of PM2.5 up to 58 μg/m^3^ was observed in 2017 compared with 2013 when the average concentration was 87 μg/m^3^. Although a significant reduction was observed, the value was still high, given that the reference value was 10 μg/m^3^ according to the WHO. However, it was observed that there were 161,613 hospitalizations for exacerbations of COPD (most patients were men over 65 years of age). Short-term exposure to air pollutants was correlated with hospital visits in the COPD emergency sections, resulting in subsequent hospitalizations and mortality (Liang et al. 2019).

In a population-based study involving 3941 nonsmoking Taiwanese adults, 791 had COPD. Exposure to PM2.5 at concentrations higher than 38.98 μg/m^3^ was associated with increased predisposition to COPD among nonsmokers in Taiwan. However, exposures to concentrations of 32.07–38.98 μg/m^3^ and 29.38–32.07 μg/m^3^ were not significant (Huang et al. 2019).

From the multitude of studies that have shown a link between COPD and air pollution, we selected this study.

### Asthma

At high concentrations, air pollutants have a direct inflammatory effect on airway neuroreceptors and the epithelium. In addition, oxidative stress has been associated with pollutant exposure (O_3_, NO_2_, PM2.5) (Johannson et al. 2014). Airway inflammation can be induced by specific pollutants (O_3_, NO_2_, PM2.5), while airway hyperresponsiveness can be induced by O_3_ and NO_2_ (Johannson et al. 2014) (Kravchenko and Lyerly 2018).

The EGEA study conducted on 204 adult asthmatic patients revealed important data about the role of oxidative stress in the association between air pollution and asthma. The levels of fluorescent oxidation products (FlOPs), an oxidative stress-related biomarker, increased with PM10 and O_3_, and the risk of persistent asthma increased with plasma FlOP levels (Havet et al. 2019).

Air pollution represents one of the most important factors aggravating asthma in children, with higher incidences in European and Caribbean regions (Cadelis et al. 2014)(Akpinar-Elci et al. 2015). One of the contributing factors is Saharan dust (Gyan et al. 2005). Saharan particles are composed of mineral origins. They are composed of a multitude of particles, such as clay, quartz, silicon oxide, and carbonates. They are lined with organic matter represented by bacteria and spores or pollen grains. Saharan dust contains PM10 and PM2.5–10, which can further predispose to an increase in visits to the emergency service for patients aged 5–15 years (Cadelis et al. 2014).

In a retrospective study of 5 years conducted by Muge Akpinar-Elci, the relationship between Saharan dust and exposure, climatic variables, and asthma was analyzed. There were 4411 recorded asthma-related emergency visits, and variation in asthma was correlated with dust concentration (Akpinar-Elci et al. 2015). Additionally, in a study conducted by Cadelis et al., there were 836 visits for asthma, with 514 boys and 322 girls (Cadelis et al. 2014).

In a study that took place in 10 European cities, the incidence of asthma among children was 14%, and after exposure to air with polluting compounds from road traffic, children with exacerbated asthma constituted 15% of the cohort. Asthma symptoms have been correlated with short-term exposure to ambient PM2.5 and PM10 in prospective cohorts, particularly in children with allergic sensitivity (Guarnieri and Balmes 2014). In a cohort study conducted by Bowatte et al., exposure to traffic-related air pollution (TRAP) was associated with both persistent and new-onset asthma in adults. Living < 200 m from a major road was correlated with greater odds of new asthma for middle-aged persons who never had asthma by 45 years. Asthmatic participants at 45 years had an increased risk of persistent asthma up to 53 years if they were living < 200 m from a major road compared with asthmatic participants living > 200 m from a major road (Bowatte et al. 2018).

### Lung cancer

Lung cancer represents one of the most common types of cancer and has a poor prognosis. The most important risk factor incriminated in developing lung cancer is active smoking, but exposure to environmental occupational carcinogens, residential radon, and passive smoke is also recognized as risk factors (Raaschou-Nielsen et al. 2013).

Although the association between lung cancer and long-term exposure to air pollution has been clarified, the link between lung cancer mortality and short-term exposure to air pollution remains unknown. The number of lung cancer cases is expected to increase due to continuous exposure to air pollution in regard to massive industrialization, an aging population and constant high smoking prevalence (Wang et al. 2019d). PM2.5, PM10, and O_3_ contribute to oxidative stress within the respiratory system and therefore potentially facilitate pulmonary inflammation and could initiate or promote the mechanisms of carcinogenesis (Xing et al. 2019). PM2.5 is considered the most relevant pollutant (Hamra et al. 2014).

In 2010, cancers of the trachea, bronchial tree, or lungs attributable to exposure to PM2.5 accounted for approximately 7% of total mortality. The mechanisms that have been incriminated in the association between PM2.5 and lung cancer include DNA deterioration and cell cycle changes (Longhin et al. 2013). PM2.5 was also related to increased production of reactive oxidative species.

A correlation was observed between the aerodynamic diameter of the fine particles in the medium ≤ 2.5 (PM2.5) and the incidence and mortality from lung cancer. Based on a meta-analysis of 18 studies, the correlation between PM2.5 and PM10 and the incidence and mortality of lung cancer were studied. Following the analysis, it was observed that the meta-relative risk was 1.09 for lung cancer related to PM2.5 and 1.09 for PM10. Additionally, the risk of adenocarcinoma associated with PM 10 was 1.29, while for PM2.5, it was 1.4. These results can help us better analyze the pathology of bronchopulmonary cancer in connection with air pollution (Hamra et al. 2014).

In the Ahsmong-2 study, it was shown that for each 10 μg/m^3^ increase in ambient PM2.5, the incidence of lung cancer increased, although the individuals from the study were exposed to low levels of ambient PM2.5 and had never smoked. The percentage was higher for individuals who had a longer period of residence and who had spent more than 1 h/day outside. The predominant type of cancer was adenocarcinomas, with a percentage of 66.4% (Gharibvand et al. 2017).

Wang et al. suggested that the carcinogenic effects of PM2.5 vary by gender as well as by the environment in which individuals live, i.e., rural or urban. It has also been observed that younger people have a lower sensitivity than elderly people. For individuals in rural areas, it was observed that with a growth level of the average concentration of PM2.5 by 10 μg/m^3^, the incidence and mortality from lung cancer were 15% and 23% among men, compared with 22% and 24% among women, respectively. Thus, following this study, the results showed that women have a meaningful risk of developing lung cancer in correlation to PM2.5 exposure (Wang et al. 2019a).

### Idiopathic pulmonary fibrosis

Idiopathic pulmonary fibrosis (IPF) is defined as a specific form of chronic, progressive fibrosing interstitial pneumonia of unknown cause, occurring primarily in older adults, and limited to the lungs. It is a progressive lung disease with a complex etiology (Johannson et al. 2018) characterized by progressive worsening of dyspnea and lung function and is associated with poor prognosis (Raghu et al. 2011).

There are not enough studies to certify the effects of air pollution on interstitial lung disease. However, in a study conducted by Johansson, it was shown that acute exacerbations of IPF were associated with an increase in the mean level, the maximum level, and the number of exceedances above accepted standards of O_3_ and NO_2_ (Johannson et al. 2014). Of the six criteria regulated by the US Environmental Protection Agency, particulate matter (PM), ground level (O_3_), and NO_2_ were strongly related to adverse respiratory effects (Johannson et al. 2015).

One potential mechanism by which ambient air pollution may cause interstitial lung disease is oxidative stress through the generation of excess reactive oxygen species (ROS), such as radical hydroxide and superoxide anion. IPF patients exhibit evidence of reduced antioxidant capacity, suggesting that they may have an increased vulnerability to excess ROS (Johannson et al. 2015). Another explanation for the progressive evolution of the disease was highlighted by the study of Winterbottom et al. on 135 subjects evaluated between 2007 and 2013. The results showed a strong association between PM10 levels and a decrease in forced vital capacity (FVC). With each μg/m^3^ increase in PM10, there was an additional 46 cc/year decline in FVC. The significant relationship observed between the exposure to coarse (PM10) and the decline rate of FVC was not reported for PM2.5, showing an inverse relationship between the diameter size of the particle and penetration into the airways. Each 5 μg/m^3^ increase in ambient PM2.5 concentration at residences corresponded with an additional 1.15 L/year increase in oxygen use on the 6-min walking test (6MWT) (Winterbottom et al. 2018). The results are equivocal, because the study conducted by Kerri A. Johannson et al. showed that PM10, PM2.5, and NO2 were associated with reduced lung function, but the changes were independent of air pollution exposure (Johannson et al. 2018).

There were no significant relationships between mean weekly change in air pollutant levels and concurrent weekly changes in forced vital capacity (FVC), forced expiratory volume during the first second (FEV1), University of California San Diego Shortness of Breath Questionnaire (UCSD-SOBQ), or visual analog scale (VAS) scores. Nevertheless, regarding the duration or interval of assessment periods, there was no significant association between the mean level of air pollutants and subsequent changes in lung function. Additionally, neither higher cumulative mean exposures nor maximal exposures to air pollution were associated with a more rapid decline in FVC or FEV1 over the study period. However, in patients with IPF, average exposures to NO_2_, PM2.5, and PM10 were associated with lower FVC, indicating that air pollution may influence the severity of disease in some individuals (Johannson et al. 2018).

In a study with 436 patients performed by Johannson et al., 75 of them had at least one acute exacerbation, and a subgroup of 13 patients had more than one exacerbation. During the exposure period, acute exacerbation of IPF was correlated significantly with increased mean rates, maximum levels, and amounts of O_3_ and NO_2_ exceedances. Increased exposure to O_3_ and NO_2_ over the preceding 6 weeks was associated with a high risk of acute exacerbation of IPF. This suggests that air pollution could be correlated with the development of this clinically significant event. At the same time, there were no consistent relationships between PM10, SO_2_, or CO and acute exacerbation of IPF compared with NO_2_, O_3_, and PM2.5 (Johannson et al. 2014).

### Respiratory infections

In the cross-sectional study of Nsoh et al., 1849 patients diagnosed from January 2013 to April 2016 with acute respiratory infections (ARIs) were registered. Of the selected patients, more than 70% used at least one form of solid fuel for cooking. In poorly ventilated homes, the impact of this exposure was irritation of the respiratory tract and eyes and an increased risk of cancers related to long-term inhalation of this poor-quality air. The probability of developing ARI was 3.62 times higher for people who were exposed to cooking indoors than for those who were not exposed. Additionally, the chances of developing ARI were 1.91 times higher for those exposed to open fire than for those who were not exposed. Thus, PM2.5 values were 13.2 times higher than what the WHO recommends. Dry weather and dust also increase the risk of developing ARI (Nsoh et al. 2019).

A study conducted in China found that with increasing concentrations of PM2.5 and PM2.5–10 compounds, the number of hospital visits for upper airway infections and pneumonia meaningfully increased. The increase in the average concentration, which accumulated over 6 days, was 10 μg/m^3^ (Z. Zhang et al. 2019).

Zheng P et al. constructed a seasonal model of cases of respiratory infections, revealing a higher preponderance in the period with lower temperatures. While children aged 5–14 years had a higher chance of developing acute respiratory infections (55.1%), those under 5 years had a higher chance (60.5%) of developing lower acute respiratory infections. The concentrations of air pollutants PM10, NO_2_, and SO_2_ exhibited lower values in the warmer period. Young children have a higher degree of susceptibility than older individuals due to their less developed immune system, tighter airways, higher frequency of respiration, and higher long-term exposure to air pollutants of the lower respiratory tract. Due to the excessive use of coal for heating during the colder season, winds also contribute to increased concentrations of air pollutants (P. Zheng et al. 2017).

### Bronchiectasis

Bronchiectasis is defined as inappropriate and permanent dilatation of the bronchi. It is a chronic respiratory disease, with many patients having frequent exacerbations. Due to their exacerbations, lung function will subsequently decrease, furthermore, increasing mortality (Garcia-Olivé et al. 2018).

Infectious pathogens are often incriminated in the majority of bronchiectasis exacerbations, but frequently, no pathogen can be identified. In a study conducted by C. Pieter Goeminne et al. on 432 patients diagnosed through high-resolution computed tomography (HRCT) and clinically confirmed bronchiectasis, for a 10 μg/m^3^ increase in PM10 and NO_2_, the chance of developing an exacerbation in that same day increased by 4.5% and 3.2%. In total, 11.2% for PM10 and 4.7% for NO_2_ were the risk of having an exacerbation for a 10-μg/m^3^ increase in the concentration of air pollutants. Subanalysis showed considerably higher relative risks through spring and summer due to increased expected outdoor air pollution exposure (Goeminne et al. 2018).

Additionally, in a retrospective observational study conducted in Badalona, SO_2_ was considerably related to an increase in the hospitalization number (Garcia-Olivé et al. 2018). Through our search of the literature, we noticed that there are few studies on the connection between air pollution and bronchiectasis.

### Tuberculosis

According to the WHO, in the 2019 Global Tuberculosis Report, approximately 10 million people worldwide fell ill with tuberculosis in 2018, and it is the leading cause of a single infectious agent. Worldwide, tuberculosis is considered to be the 10th leading cause of death (WHO-Global Tuberculosis Report 2019 n.d.) Tuberculosis (TB) is a disease whose prevalence has been associated with socioeconomic risk factors that has a stronger association with urban settings, where there is greater exposure to air pollution (Jassal et al. 2012).

There is a direct correlation between air quality and tuberculosis incidence. Precipitation, atmospheric pressure, and relative humidity affect the incidence of tuberculosis by indirectly reducing the quantity of inhalable PM and SO_2_ concentrations. On the other hand, wind plays a major role by increasing the incidence of tuberculosis by spreading pathogens (Zhang and Zhang 2019). Fine particulate matter and traffic-related air pollution might be associated with an increased risk of developing tuberculosis. This association is not due to direct exposure but rather to the impairment of the individual’s immunity (Lai et al. 2016). Popovic et al. showed in a systematic review that the pollutant most frequently associated with tuberculosis is PM2.5 (Popovic et al. 2019).

The Chengdu study also documented that exposure to ambient PM10, NO_2_, and SO_2_ was linked to increased tuberculosis morbidity in China, but the lag time was 28 days for PM10 and 5 days for SO_2_ and NO_2_, which can only be attributed to short-term effects (Zhu et al. 2018). Another study conducted by Lai et al. highlighted that an increased risk of active tuberculosis is related to exposure to fine particulate matter PM2.5. Furthermore, traffic-related air pollution, including nitrogen dioxide, nitrogen, and carbon monoxide, was associated with tuberculosis risk. On the other hand, PM10 was not linked with active tuberculosis, and O_3_ was inversely associated with the risk of TB (Lai et al. 2016).

Similar to the last results, O_3_ levels could not be significantly correlated with acid fast bacilli (AFB)-positive smears in the retrospective study of Jamal et al. Medical records of 196 individuals diagnosed with TB positivity at Los Angeles County and University of Southern California Medical Center Hospital were analyzed. A total of 111 had smear positivity, while 85 had smear negativity. There was a significant correlation in single pollutant models analyzing PM2.5 levels and smear-positive TB (Jassal et al. 2012).

The link between PM2.5 concentration, notably a 10 μg/m^3^ increase in PM2.5 levels, and active TB was also noted in a study conducted from 2014 to 2017 in Lianyungang. For the single-pollutant model, the association between a 10-μg/m^3^ increase in PM10 concentration and SO_2_ concentration and active TB was significant. Additionally, a potential correlation between relatively long-term outdoor exposure to PM2.5, PM10, SO_2_, and NO_2_ and active TB was observed in the time-series study conducted in the northeastern region of Jiangsu Province, China. In the multipollutant models, ambient PM10 and NO_2_ remained significant, and the association was not altered in subgroups of different genders or ages (Li et al. 2019).

In addition, exposure to pollution over different periods of time may be associated with drug resistance. Exposure to PM2.5, PM10, O_3_, and CO has been associated with drug-resistant TB, including first-line monodrug resistance, polydrug resistance, and multidrug resistance (MDR), in both single- and multiregression models. In the retrospective study of Yao et al., conducted in Jinan city, China, from January 1, 2014 to December 31, 2015, 752 new culture-confirmed TB cases reported in TB prevention and control institutions of Jinan were included. The results showed significant monodrug resistance, and polydrug resistance increased the risk for ambient PM2.5, PM10, O_3_, and CO exposure. The most significant association for PM2.5 was noticed at 540 days of exposure, for O_3_ was noticed at 180 days of exposure, and for PM10 and CO, it was noted from 90 to 540 days of exposure. Of the 752 cases, 18.8% were first-line drug-resistant cases with streptomycin having the highest rate of resistance (15.3%), 13% were second-line resistant, fluoroquinolones having the highest rate of resistance (11.3%), 12.3% were resistant to more than 1 drug but not MDR, and 3.3% were MDR-TB (Yao et al. 2019).

*NO*_*2*_ nitrogen dioxide, *SO*_*2*_ sulfur dioxide, *PM2.5* particulate matter with diameter < 2.5μm in diameter, *PM10* particulate matter with diameter < 10μm in diameter, *O*_*3*_ ozone, *CO* carbon monoxide, *COPD* chronic obstructive pulmonary disease, *IPF* idiopathic pulmonary fibrosis

### Animal experiments

Animal experiments have opened up new perspectives on air pollution (Edwards et al. 2020). Air pollution contributes to increased inflammation. When polluted air is inhaled, its first stop is the lungs. This is where oxidation-reduction first occurs (Gangwar et al. 2020). Oxidative stress arises from altering the balance between oxidants and antioxidants. Altering this balance will increase oxidative stress and cause the increase of lung pathologies through promoting inflammation of the airways. PM consists of a number of components capable of generating ROS, which subsequently increase inflammatory mediators in the lungs (Valavanidis et al. 2013).

In a study conducted by Edward et al., it was observed that rats exposed to TRAP, compared with those not exposed, exhibited increased gene expression changes related to oxidative stress, inflammation, aging, and fibrosis in the heart (Edwards et al. 2020). While Zheng et al. showed that following exposure to tracheal diesel particles, mice presented an increase in transient oxidative stress in the lungs, Sun et al. showed that PM2.5 accentuates the degree of atherosclerosis, degrades vasomotor tone, and determines vascular inflammation in mice that have been chronically exposed to low concentrations of PM2.5 (Q. Sun et al. 2015) (X. Zheng et al. 2019) (Gangwar et al. 2020). Rats that were exposed to ozone showed 8-hydroxy-2′-deoxyguanosine (8-OHdG) and heme oxygenase-1 (HO-1) in macrophages, developing rigid lungs with reduced function (Sunil et al. 2013) (Valavanidis et al. 2013).

Fibrosis and reversible cardiac dysfunction were observed in mice after intratracheal exposure to PM2.5 (Gangwar et al. 2020). Oxidative stress in the myocardium is increased in those exposed to ultrafine particles (Cozzi et al. 2021). Qin and all demonstrated that after intratracheal exposure to PM2.5, the most sensitive were the extremes of age compared with adult animals, developing heart dysfunction and reversible fibrosis (Qin et al. 2018) (Gangwar et al. 2020). Cozzi et al. showed that myocardial damage in mice exposed to ultrafine particles is double that in those not exposed (Cozzi et al. 2021).

### General information

When the level of pollution is high, informing the population should be a priority. This information should be free and easy to access so that outdoor activity is reduced during periods with higher air pollution (Tiotiu et al. 2020).

Air quality alerts are beneficial to the population. The population is notified when air quality alerts occur (Wen et al. 2009). In a study conducted by Graff and Neidell, it was found that when the population was alerted by smog alerts, outdoor physical activity on visits to the Griffith Park Observatory and Los Angeles Zoo decreased by 8% and 15%, respectively. However, when alerts were repeated on days 2 and 3, people did not take into account the smog alerts, with values of 0% and 5%, respectively (Graff and Neidell 2009). These warnings should be repeated as often as possible and should be of particular interest for patients with cardiopulmonary pathology, as well as healthy patients who may subsequently develop chronic diseases (Wen et al. 2009).

From a real estate point of view, both large cities and those with fewer inhabitants should be channeled on development so that the degree of pollution does not affect the quality of life of the population. This should be done from the beginning and not developed later, after the urbanization plan has been made. This would help reduce air pollution from the start. Public institutions, as well as the community, must contribute to reducing the degree of pollution. However, although institutions should play a key role in reducing pollution, it can also be reduced by individual freewill (Carlsten et al. 2020).

As medical staff inform asthmatics to avoid aeroallergens, patients with chronic cardiopulmonary disease should also be informed by the degree of air pollution and how it may affect their health. Otherwise, they may develop new symptoms or experience worsening of pre-existing symptoms. The air quality index (AQI) should be consulted frequently by patients to cancel outdoor activities when air quality is poor (Shofer et al. 2007) (Wen et al. 2009).

The use of masks helps reduce the degree of inhalation of noxious substances. However, not all masks are equally effective, and this depends on both the type of mask and the filter it has (Carlsten et al. 2020). In a study conducted by Shakya et al., masks made from material were beneficial to a low degree in protecting particles with a diameter of 2.5 μm, while surgical masks were more effective. The most efficient in eliminating most tested particles was N95 masks. The material masks have a higher comfort but are much weaker than N95 masks (Shakya et al. 2016).

## Conclusions

**Fig. 1:**
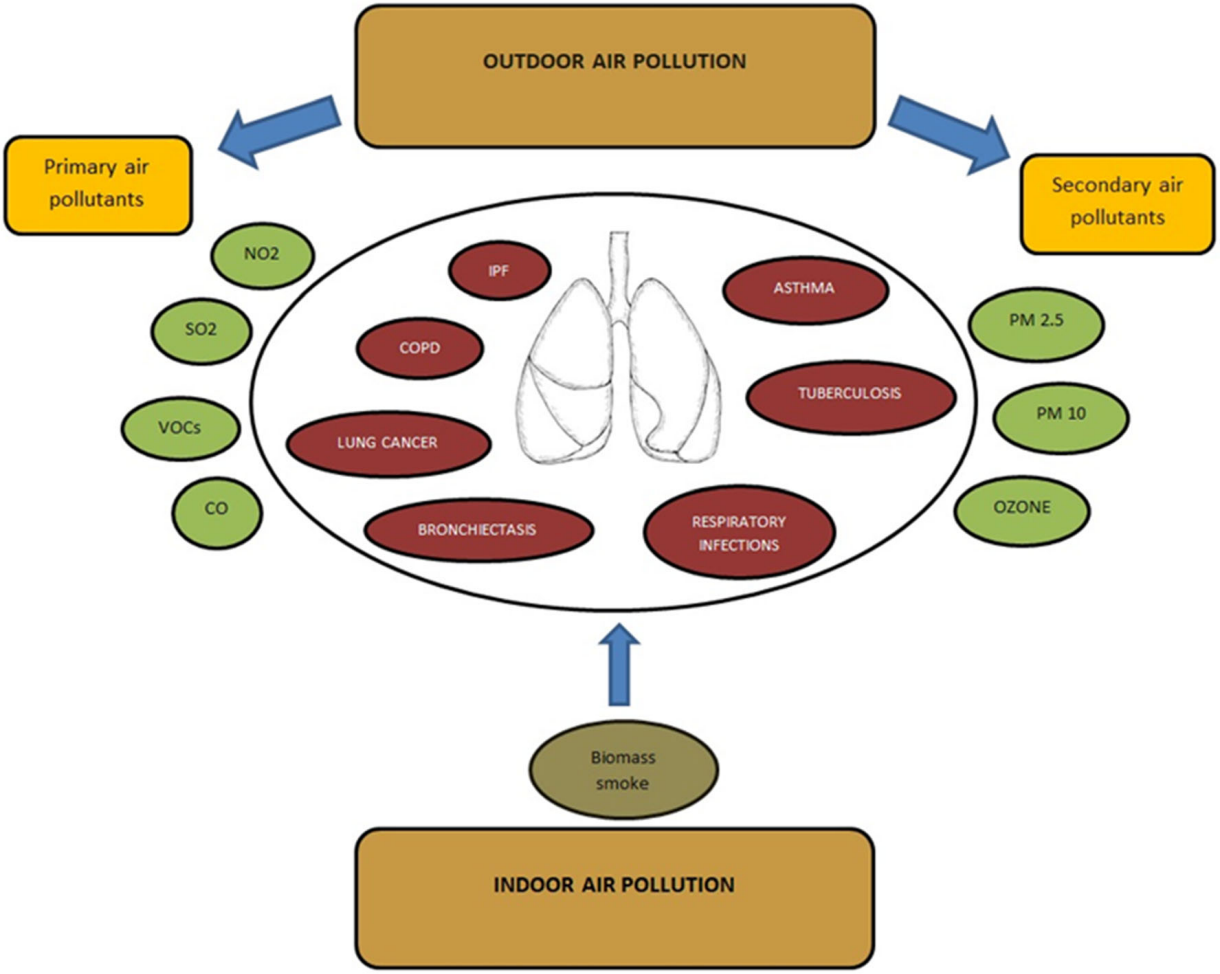
NO^2−^ nitrogen dioxide, SO_2_ sulfur dioxide, VOCs volatile organic compounds, CO carbon monoxide, PM2.5 particulate matter with diameter < 2.5 μm in diameter, PM10 particulate matter with diameter < 10 μm in diameter

Today, although we know the impact of pollution on the respiratory system, we have tried to describe up-to-date information on how pollution affects the respiratory system and the pathologies associated with it (Fig. 1). This depends on the type of pollutant, its concentration in the environment, and its size. Air pollution potentiates the increase in respiratory pathology. It is important to constantly measure the quality of the air, both in developed and less-developed countries to ensure continued improvement.

### Strength of this review

The characteristics of this review refer in particular to the lung diseases caused by air pollutants. The lung is one of the main human organs that have direct contact with the air and is able to filter inhalable pollutants. Lung damage by any other pathology corroborated with inhalable pollutants can later affect other organs and the whole body. For this reason, we considered it of major importance to classify the air pollutants and to present how each pollutant influences lung pathologies and can later affect the whole body.

## Abbreviations

WHO: World Health Organization
SO2: sulfur dioxide
NO2: nitrogen dioxide
CO: carbon monoxide
VOCs: volatile organic compounds
IARC: International Agency for Research on Cancer
O3: ozone
PM: particulate matter
PM2.5: particulate matter with diameter < 2.5 μm in diameter
PM10: particulate matter with diameter < 10 μm in diameter
COPD: chronic obstructive pulmonary disease
FIOPs: fluorescent oxidation products
TRAP: traffic-related air pollution
IPF: idiopathic pulmonary fibrosis
ROS: reactive oxygen species
FVC: forced vital capacity
6MWT: 6-min walking test
FEV1: forced expiratory volume during the first second
UCSD-SOBQ: University of California San Diego Shortness of Breath Questionnaire
VAS: visual analog scale
ARI: acute respiratory infections
HRCT: high-resolution computed tomography
TB: tuberculosis
AFTB: multiresistance drug resistance
